# From Psoriasis to Psoriatic Arthritis: Insights from Imaging on the Transition to Psoriatic Arthritis and Implications for Arthritis Prevention

**DOI:** 10.1007/s11926-020-00891-x

**Published:** 2020-05-16

**Authors:** Alen Zabotti, Ilaria Tinazzi, Sibel Zehra Aydin, Dennis McGonagle

**Affiliations:** 1 Department of Medical and Biological Science, Rheumatology Clinic, University of Udine, Udine, Italy; 2 Unit of Rheumatology, IRCSS Ospedale Sacro Cuore Don Calabria, Negrar, Verona, Italy; 3 Faculty of Medicine, Division of Rheumatology, University of Ottawa, the Ottawa Hospital Research Institute, Ottawa, Canada; 4 Leeds Institute of Rheumatic and Musculoskeletal Medicine, University of Leeds, Leeds, UK

**Keywords:** Psoriatic arthritis, Psoriasis, Ultrasonography, Arthralgia, Prevention

## Abstract

**Purpose of Review:**

To describe the recent advances in the field towards the prevention and early recognition of Psoriatic Arthritis (PsA).

**Recent Findings:**

Defining the preclinical phase of PsA remains challenging since up to 50% of subjects with psoriasis have subclinical imaging enthesopathy, but many of these do not progress to PsA. Nevertheless, there is evidence that subjects with subclinical imaging enthesopathy are at increased risk of developing PsA. In recent years, it has been shown that both PsA and anti-citrullinated protein antibodies (ACPA) positive rheumatoid arthritis (RA) are characterized by a subclinical phase of non-specific or brief duration arthralgia with shared imaging features accounting for joint symptomatology. Sonographically determined tenosynovitis and enthesitis are the key imaging features present in non-specific PsO arthralgia that are at risk of future PsA development. Furthermore, the early phases of PsA are complicated by factors including body mass index (BMI), which is a risk factor for PsA, but BMI is also associated with imaging abnormalities on enthesopathy. Fully disentangling these clinical and imaging factors will be important for enrichment for imminent PsA so that disease prevention strategies can be investigated.

**Summary:**

Psoriasis patients with arthralgia have a higher prevalence of tenosynovitis and imaging enthesopathy is at higher risk of transitioning to overt PsA.

## Introduction

Anti-citrullinated protein antibodies (ACPA) positive patients and subjects with psoriasis are two populations at higher risk of respectively developing rheumatoid arthritis (RA) and psoriatic arthritis (PsA) [1, 2]. However, unlike preclinical RA where therapy may be potentially toxic in an otherwise asymptomatic patient, the presence of psoriasis, that is often extensive sets up a scenario whereby dermatological directed therapy could prevent arthritis evolution at “no extra cost”. There is a strong link between subclinical enthesopathy and psoriasis, but how this translates into frank PsA development remains uncertain [3, 4]. Nail disease in PsA is a strong predictor of future PsA development, and it has been shown that the nail is anchored to the skeleton and that subclinical imaging enthesopathy is common in psoriasis subjects with nail disease but without psoriasis [5, 6]. In animal models of PsA, the earliest lesion is at the enthesis [7, 8]. Collectively these findings indicate that imaging of the joints in psoriasis and especially enthesopathy may be relevant for predicting PsA development and hence facilitate timely intervention. In this review, we discuss the role of imaging as possible biomarker of PsA development and as a tool to better understand the mechanisms of transition from psoriasis to PsA.

## The Transition to Psoriatic Arthritis

PsA mostly develops in patients with an established diagnosis of psoriasis, the diagnosis of psoriasis occurring after the onset of arthritis only in 15% of PsA cases [9]. The incidence of PsA after the onset of psoriasis increases with time, reaching to 20% after 30 years [10]. It is important to identify patients at risk of developing PsA which can enable targeting prevention. Nail, scalp, and inverse psoriasis and the severity of the cutaneous disease are the clinical features with higher risk of PsA development [5, 9]. Among comorbidities, obesity is associated with PsA development [11, 12]. There is also a link between the magnitude of body mass index (BMI) elevation and future PsA risk [12]. From a genetic point of view, as for RA, a first degree relative with arthritis could contribute to increase this risk [13]. Eder et al. highlighted that arthralgia in psoriasis females is a strong predictor of PsA development [14••]. We recently confirmed these results in a longitudinal study in which psoriatic patients with arthralgia (PsOAr) were more prone to develop PsA compared to psoriatic patients without musculoskeletal complaints (PsO) [15••]. These studies bring attention to symptoms referred by patients in their preclinical phases of PsA as possible markers of imminent PsA development.

It has recently been proposed that there are three clinically quiet stages after psoriasis onset and before clinically detected PsA [16••]. These are as follows: (a) preclinical phase which is characterized by aberrant activation of the immune system which may originate from the skin, intestinal mucosa or the entheses. (b) Subclinical PsA phase with soluble biomarkers and imaging findings with no clinical symptoms. (c) Prodromal PsA in which patients have arthralgia and fatigue with no synovitis and or enthesitis on physical exam yet *(*Illustration 1*)*. In our opinion, when PsA develops after acute joint injury or after infection, this model could not be necessarily applied. In this article we summarize the literature on imaging in psoriasis and PsA and point out how the prodromal phase of PsA actually is associated with tenosynovitis on imaging. We also discuss the possibility of PsA prevention based on the emerging imaging data.

**Illustration 1.**
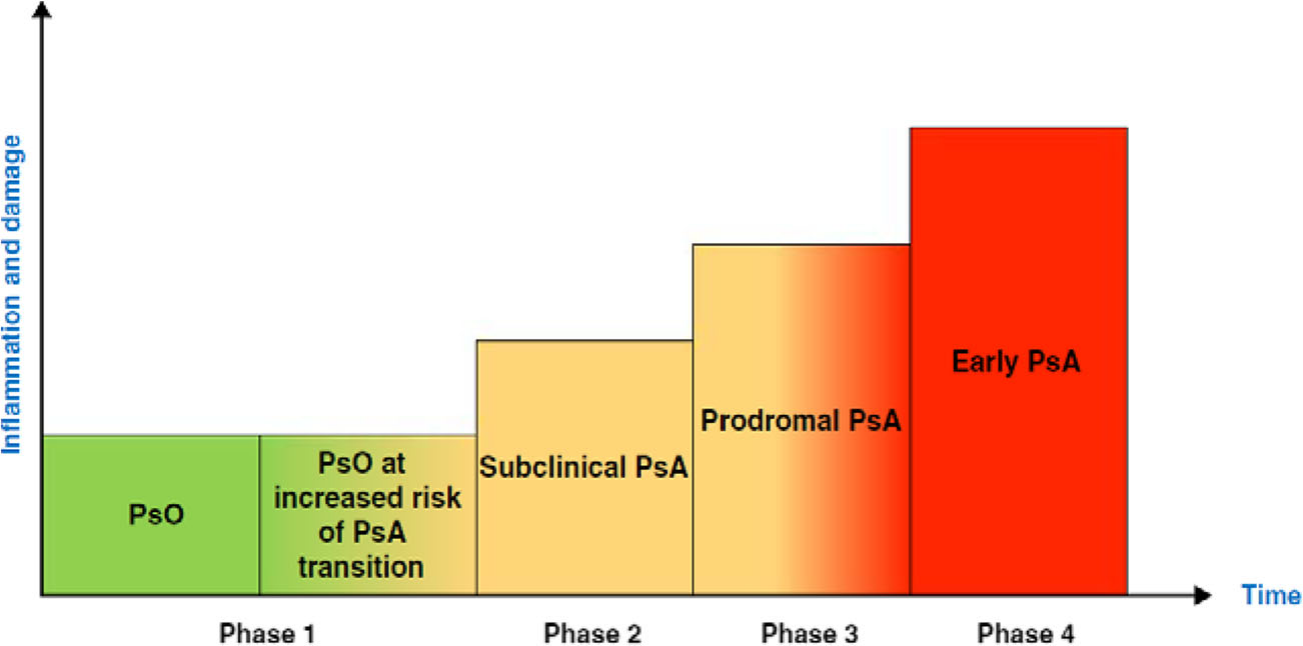
Transition phases from psoriasis to early PsA

## Subclinical PsA

### Imaging Features in Psoriasis Patients without Musculoskeletal Complaints

**Fig. 1:**
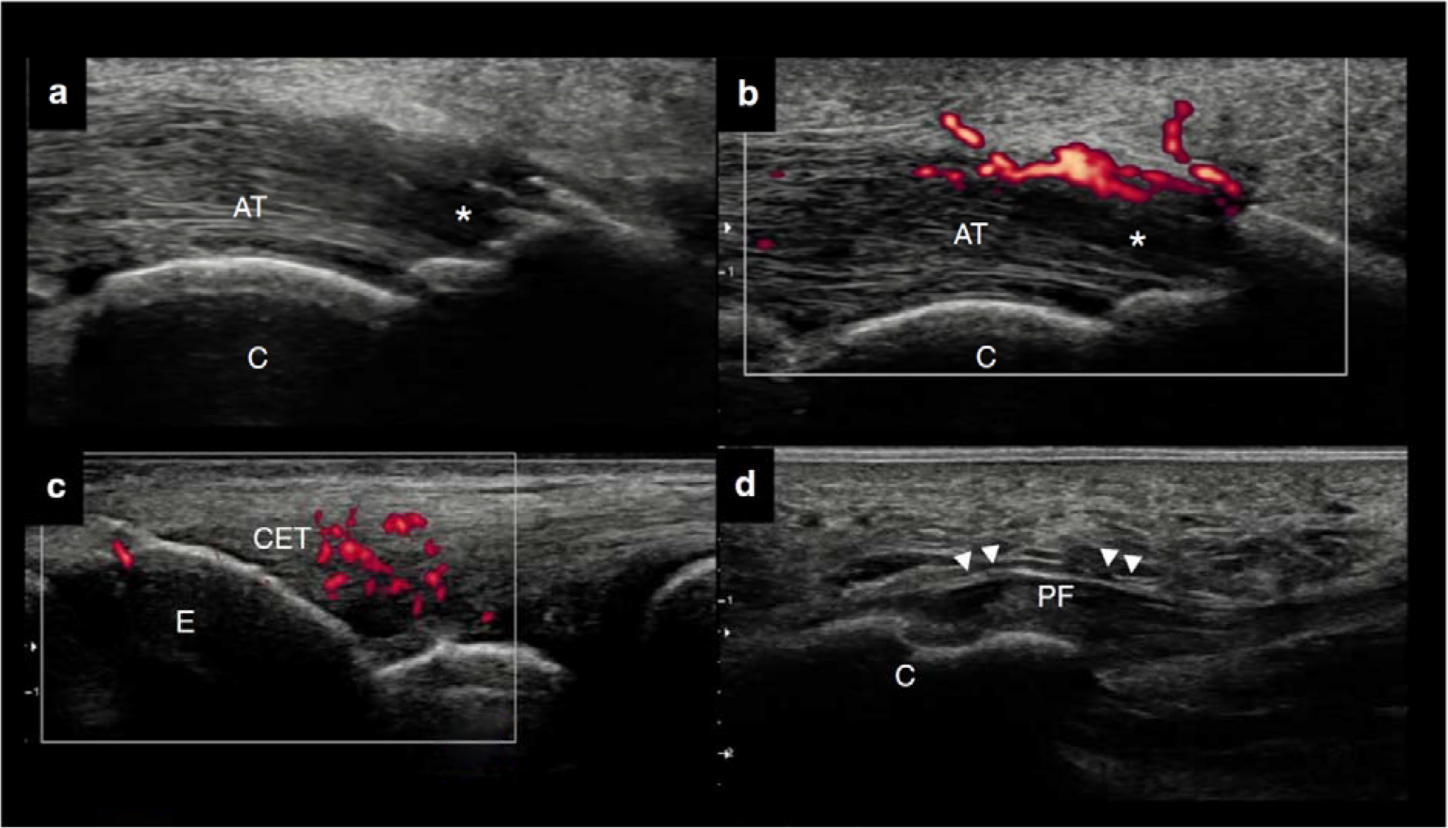
**a–b** Dorsal scan of the Achilles tendon. **a–b** Longitudinal view. Active enthesitis with loss of the tightly packed echogenic lines (white asterisks) and concomitant PD signal at bone insertion in a psoriatic arthralgia patient. **c** Lateral scan on the elbow. Active enthesitis of the common extensor tendon enthesis in an early psoriatic arthritis. **d** Plantar fascia. Thickening of the plantar fascia in a psoriasis patient. *AT*, Achilles tendon; *C* calcaneus; *CET* common extensor tendon enthesis; *PF* plantar fascia

High-frequency sonography is an imaging technique to evaluate skin, nail, joints, and entheses in psoriatic patients that can allow detection of subclinical signs of PsA *(*Fig. 1*).* Many studies had compared the ultrasound findings of the large entheses (mostly the weight bearing lower limb entheses) in PsO patients and healthy controls (HCs), and the majority of them showed PsO patients had more entheseal inflammation on ultrasonography (US), despite being asymptomatic [17]. *Simon* et al. showed that the number of enthesophytes detected by high-resolution peripheral quantitative computed tomography (HR-pQCT) was significantly greater compared to HCs [18]. Furthermore, they found that duration of skin disease influenced enthesophytes burden in PsO [18]. Recently, also subclinical synovitis was found significantly more frequent in PsO than in HCs [19, 20]. Currently, if the imaging criteria of subclinical inflammation are used, up to half of PsO patients could be classified as subclinical PsA [20] *(*Illustration 1*).* The identification of specific imaging lesions, associated with overt clinical PsA development, will be the challenge for the coming years, and this step will be essential to stratify correctly PsO patients at risk for PsA development [4, 6]. Subclinical inflammation in PsO patient should be assessed not only at enthesis and synovial level but also in the vessels allowing to prevent cardiovascular events. Metabolic imaging studies, such 18F-fluorodeoxyglucose positron emission tomography-computed tomography (18F-FDG PET/TC), have documented capability to assess vascular disease and the ability of anti-TNF therapy to reduce vascular uptake after a year of therapy [21]. Few studies were designed to study the increased cardiovascular risk in PsO using magnetic resonance imaging (MRI) to assess aortic wall thickness or using US to assess carotid intima-media thickness [22, 23].

### Non-Specific Joint Pain in Psoriasis or Prodromal PsA

#### Imaging Features in Psoriasis Patients with Arthralgia

**Fig. 2:**
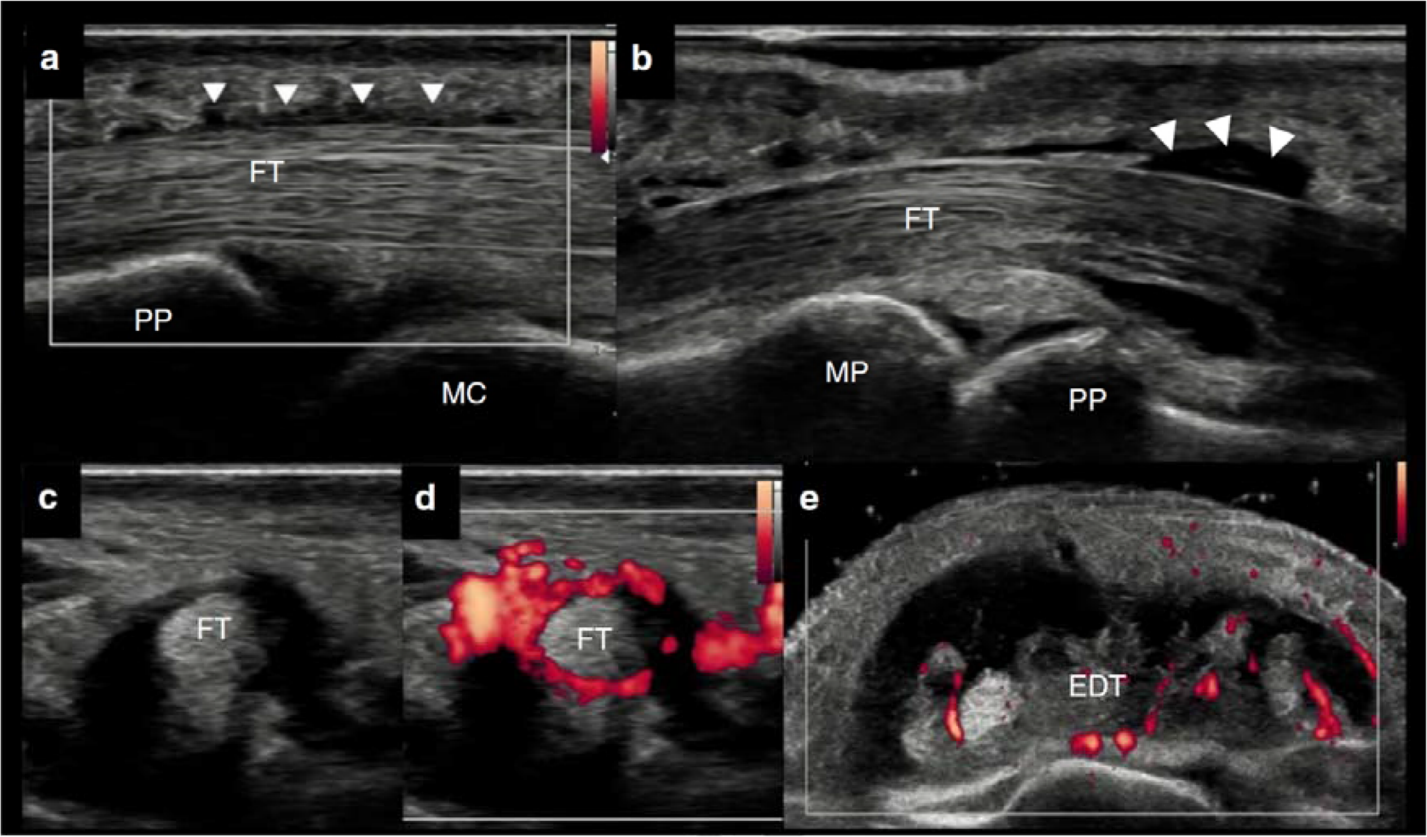
**a–d** Volar scan of the digit of the hands by ultrasonography. **a–b** Longitudinal view. Mild tenosynovitis (white arrowhead) of the flexor tendon (FT) in a psoriasis patient with arthralgia. **c–d** Transverse view. Active tenosynovitis of the flexor tendon in an early psoriatic arthritis. Dorsal scan of the wrist. **e** Active tenosynovitis of the extensor digitorum tendon (EDT) in an established psoriatic arthritis

The last transition phase from psoriasis to PsA, named prodromal PsA, seems to be clinically characterized by non-specific musculoskeletal symptoms [14••, 16••] *(*Illustration 1*)*. The high risk and the likely short time before progression to clinically evident PsA give the opportunity to visualize the possible mechanisms of expansion of inflammation from the skin to joints or the evolution from a subclinical form to a clinically manifested disease. In a clinical and sonographic study on PsOAr, sonographically determined tenosynovitis was the most significant contributor to symptoms in PsOAr; indeed, sonographic investigation revealed tenosynovitis, mainly of the flexor tendon of the hands, in 29.5% PsOAr patients compared to 5.3% in PsO patients [15••] *(*Fig. 2*).* Active enthesitis and synovitis, albeit commoner in PsOAr, did not reach a significant difference between PsOAr and PsO cohorts but in the longitudinal part, sonographically determined enthesitis was the only US feature linked to the future evolution of PsA [15] *(*Fig. 1*).* This finding replicated the original longitudinal analysis showing that baseline enthesopathy predicted PsA [3]. Faustini et al. confirmed this relationship between subclinical inflammation detected and PsA, highlighting that patients with hands synovitis detected by MRI and arthralgia had 55.5% likelihood to develop PSA within 1 year [24]. Temporally close to clinical PsA diagnosis, psoriatic inflammatory arthralgia could be considered as an intermediate between PsOAr and very early early PsA. In the IVEPSA (Interception in very early PsA) study psoriatic patients with inflammatory arthralgia without joint swelling and with concomitant predictors of PsA (i.e. PASI > 6 or scalp psoriasis or nail involvement) were treated with anti-IL-17 for a disease interception [25••]. Baseline MRI investigation of the dominant hand revealed at least one inflammatory lesion in 83% of patients, highlighting synovitis as the most prevalent (66.7%), followed by tenosynovitis (55.6%). Micro-erosion and bone proliferation are common in psoriasis subjects without arthralgia on HR-pQCT [25••]. Therefore, it is no surprise that bone proliferation and erosion were frequently found in psoriatic inflammatory arthralgia: at least one erosion was detected, by HR-pQCT, in 33.3% inflammatory arthralgia patients [25••].

## Psoriatic Arthritis

### Early Psoriatic Arthritis

#### The Imaging of the Synovio-Entheseal Complex

**Fig. 3:**
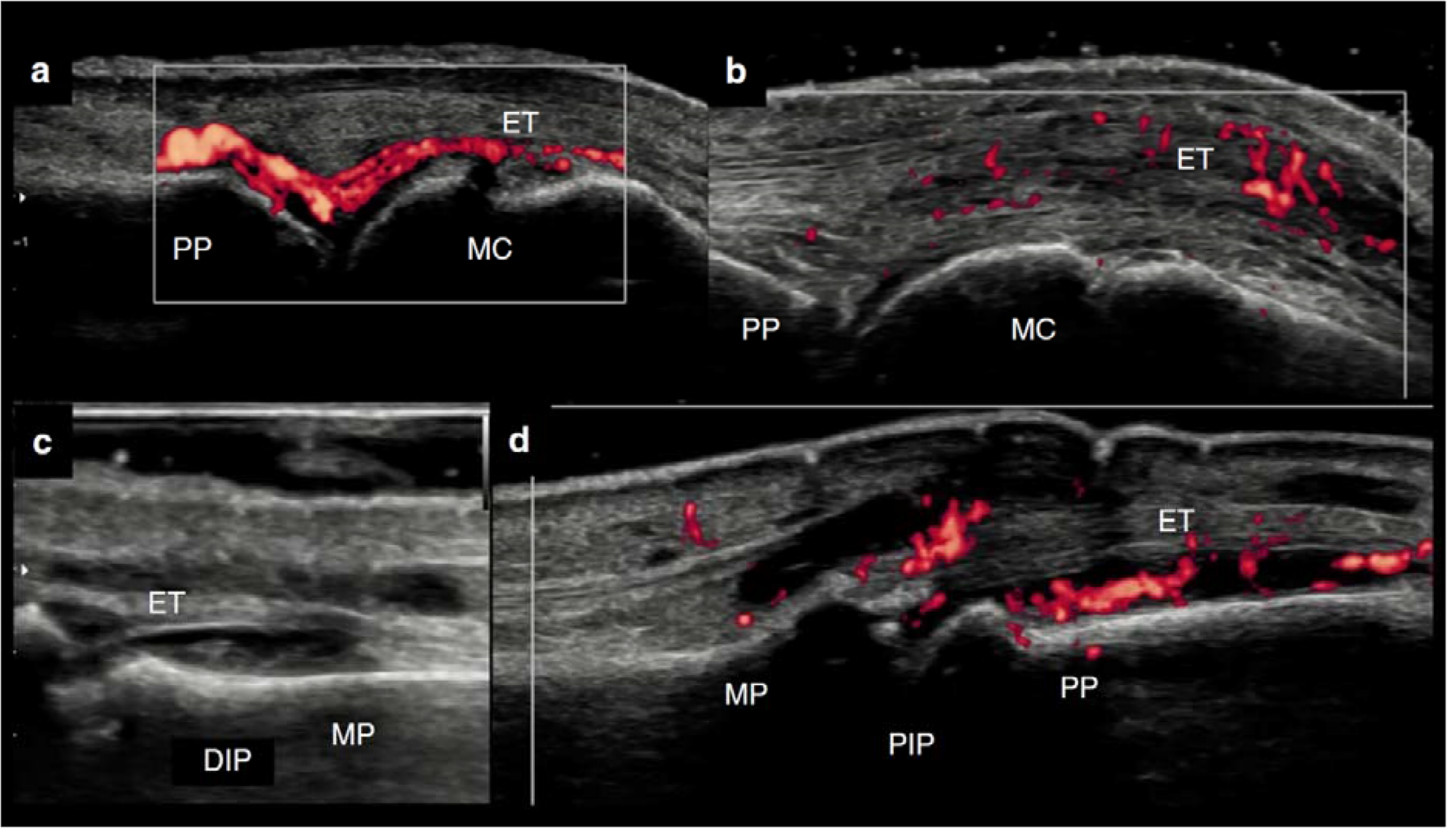
**a–b** Dorsal scan of the metacarpophalangeal joint by ultrasonography. **a** Longitudinal view. Active synovitis in an early rheumatoid arthritis (synovium-centric disease). **b** Longitudinal view. Synovio-entheseal complex (SEC) inflammation (active peritendinitis of the extensor tendon and mild active synovitis) in an early psoriatic arthritis (SEC-centric disease). **c** Dorsal scan of the distal interphalangeal (DIP) joint by ultrasonography. Longitudinal view. Synovitis of the DIP joint in an early psoriatic arthritis patient. **d** Dorsal scan of the proximal interphalangeal (PIP) joint by ultrasonography. Longitudinal view. SEC inflammation of the PIP joint (central slip enthesitis of the extensor tendon and active synovitis). *ET* extensor tendon

That the enthesis may be closely functionally integrated with the synovium was first reported in 2007, and this model of joint inflammation is supported by animal models [26, 27]. In a TNF^ΔARE^ mouse model, chronic and deregulated TNF production leading to a spondyloarthropathy phenotype with arthritis and Crohn’s-like ileitis, the onset of inflammation was seen in the entheses including the collateral ligaments of interphalangeal joints and at the synovio-entheseal complex (SEC) of the Achilles tendon [7]. The synovium was subsequently involved, followed with pannus formation, and finally involving the entire joint [7] *(*Fig. 3*).* Actually, imaging studies in early PsA confirms this hypothesis highlighting that PsA, differently from RA, seems not to be a synovial-centred-disease and presents inflammation affecting a wider area, including soft tissue and entheses, as well as synovium [28–30]. In early PsA, the sonographic detection of SEC inflammation at metacarpophalangeal joints (i.e. extensor peritendinitis with or without synovitis) and at proximal interphalangeal joints (i.e. central slip enthesitis with or without synovitis) were also described [29, 31]. Furthermore, these sonographic features are useful to differentiate PsA from RA, both in early and longstanding disease [32].

The role of mechanical strain as a trigger of the inflammatory process within the SEC was recently confirmed [33]. Cambrè et al. found that mechanical strain was an essential checkpoint for the translation of systemic immune activation into site-specific joint inflammation in mouse model [33]. This concept was highlighted also in humans: high level of occupation-related mechanical stress was indeed associated with increased radiographic peripheral joint damage among patients with longstanding PsA [34]. Furthermore, Tinazzi et al. proposed that dactylitis and tenosynovitis could be modified by early biomechanical alterations in tendon function, particularly at the pulleys level [35•]. Flexor tendon pulleys are mini-entheses that anchor the tendons to the bone and are subjected to very high physical stress. In the study the authors found that PsA patients had thicker pulleys compared with RA and HCs and this thickness was particularly relevant in dactylitis [35•]. Globally, this study seems to confirm the role of anatomical and functional entheses as initial site of inflammation and the mechanical strain as a trigger.

### Established Psoriatic Arthritis

#### The Extraarticular Manifestations and How Those Are Linked to each Other when Scanned by Imaging

Imaging elucidated the crucial role of entheses in PsA pathogenesis; the inflammation of these structures is triggered by functional overload due to overweight or chronic mechanical micro-trauma. When an inflammatory process became repeated cells of innate immunity spreads through the enthesis and other structures became involved by the inflammatory process such as joints, bursa, connective tissue, and bone. This is the case of the link between the inflammation of the nail and the distal interphalangeal (DIP) joints [36]; MRI studies suggested that the dorsal capsular enthesis of the DIP was the epicentre of the inflammatory process [37]. Patients with nail psoriasis are shown to have more extensor tendon enthesitis in the adjacent extensor tendon insertion on US as well as having more enthesitis in remote sites [6]. In obese patients the lower limbs enthesis are chronically stressed and traumatized by the overload due to overweight; it is therefore easy to observe US alterations due to chronic enthesitis like bone proliferation and erosions [17]. According to the hypothesis of obesity as a promotor of disease activity in PsA, short-term weight loss treatment was resulted with significant positive effects on disease activity in joints, entheses, and skin [38]. With imaging, BMI is found to be linked to entheseal inflammation as well as new bone formation at the peripheral entheses. The same effect on new bone formation is also seen for the axial skeleton with more syndesmophytes and a higher modified Stoke Ankylosing Spondylitis Spinal Score with increased BMI [39]. Higher enthesitis scores were also observed in inflammatory bowel disease even without symptoms of spondyloarthropathy supporting a shared pathogenic mechanism between IBD and spondyloarthritis (SpA), whereas similar scores may suggest a general impact of gut inflammation on the enthesis [40]. Subclinical bowel inflammation was correlated with the severity of spine inflammation [41]. Scarpa et al. demonstrated that bowel mucosa of patients with PsA also without bowel symptoms shows microscopic lesions supporting the pathogenetic link between skin, joints, and gut in psoriatic patients [42]. Recent research highlighted that recirculating innate immune cells from the gut to the extra-intestinal sites as possible responsible for the induction of chronic inflammatory responses in SpA patients [43]. The intestine-enthesis axis in PsA is not yet fully elucidated; whether such as micro-traumatism or a dysbiosis in the intestinal mucosa that alters the intestinal barriers is matter of ongoing studies.

## Future Steps and Research Agenda

### Imaging Towards Clinical Prevention

Since 80% of PsA patients initially have skin psoriasis, a marker that can easily be identified with inspection, there is a potential target for identifying people that has a high risk for developing PsA and treating to prevent the disease. There is a significant overlap between the treatment of psoriasis and PsA, and patients present with skin psoriasis first, therefore effective treatment of psoriasis may stop the progression to PsA. A retrospective study supported that the incidence of new symptoms leading to diagnosis of PsA is reduced if patients are on biological and conventional DMARD therapies for their skin psoriasis [44•]. In addition, biologic therapies also seem to have an effect on the disease features as none of the patients on biologics (given for psoriasis) develop dactylitis compared to 28.6% of other systemic treatments and 48.6% of none/local treatment [44•]. A recent prospective study showed that the interleukin-12 (IL-12)/IL-23 inhibition reduces the subclinical enthesopathy in psoriasis within 12 weeks which is maintained through week 52 [45••]. These data suggest that treatment of psoriasis may have disease modifying effects for PsA, and there may be a window of opportunity to prevent PsA symptoms. This aligns with studies have shown that psoriasis patients who develop PsA at follow-up have higher enthesitis scores on the ultrasound at baseline, years before developing PsA, which supports the enthesis being the key structure in PsA, and the disease may be initiated at the level of the entheses [3, 8]. There are ongoing psoriasis cohorts with extensive use of US to visualize the joints and the enthesis, which hopefully give us more insights about the pathway from psoriasis to PsA and leading to prevention rather than treatment**.**

### Better Phenotyping Patients in Clinical Practice and Trials

PsA is a very heterogeneous disease with different “seemingly unrelated” disease manifestations occurring within the same presentation. The phenotypes that have been described by Moll and Wright in 1971 nicely summarizes how patients can present, namely with axial disease, polyarticular, oligoarticular arthritis, DIP predominant disease, and arthritis mutilans, and the determination of the disease phenotypes have traditionally been based on the clinical assessment [46]. The number of tender or swollen joints determine whether it’s a poly vs oligoarticular disease, although this separation can be artificial as there may be changes in the pattern over time either due to the disease course or treatment affect [47].

There are no established definitions of axial disease in PsA or how to screen, which causes major differences across studies in terms of how often it’s seen. The inflammatory back pain criteria have been developed in Ankylosing Spondylitis and have been demonstrated to have a poorer performance in PsA which may limit their value to be used as screening tools [48, 49]. Supporting this, the percentage of patients axial PsA within PsA cohorts increases if standard imaging is done regardless of the presentation [50]. This underdiagnosed axial PsA disease (imaging findings of sacroiliitis and/or syndesmophytes with no clinical diagnosis of axial disease) has impacts on the patient outcomes with worse PROs, which cannot be detected if patients do not have standard screening [51].

The clinical trials use the commonly accepted definitions of these phenotypes, which have shown significant discrepancies with imaging modalities. For example, the vast majority of the clinical trials have only been focusing on the effectiveness of therapies on polyarticular phenotype in PsA, with few to none data on the axial disease, clinical enthesitis, or dactylitis as a primary outcome [52]. The efficacy data on features such as enthesitis or dactylitis has mostly been exploratory. In a joint-study from Ottawa, Leeds, and Toronto where US features of enthesitis were compared with clinical assessment of the same site, a relationship between clinical and sonographic findings for large entheses is found to be dependent on the anatomical site, especially the Achilles tendon and patellar tendon origin, whereas for some other sites such as the patellar ligament insertion or plantar fascia, the tenderness on physical examination did not have any corresponding feature on ultrasound [53••]. In the absence of a prospective study to compare the outcomes of patients with or without US-detected inflammation versus clinical enthesitis, it is not possible to make a statement on which one is the gold standard. However, the use of US for entheseal disease, instead of clinical examination, revealed multiple links between different disease manifestations, which could not be captured by using physical examination only, such as the link between the enthesis and the nail or the spine [6, 53••, 54]. Therefore, the use of ultrasound helps us better phenotype patients not only in clinical practice but also in clinical trials which may allow us to better understand the mechanisms causing to those phenotypes and treat.

## Conclusions

Several factors identify those psoriasis patients with the highest likelihood of progression to PsA, such as nail involvement, severe psoriasis, and obesity; however, various aspects are still required to clarify the preclinical phases of PsA, from the definition of PsA risk factors to prediction of PsA development [5, 11, 12]. These patients with clinical risk factors of PsA are often characterized by non-specific symptoms and few or any objective inflammation [14••, 15••]. In psoriasis patients with no specific features on clinical examination, imaging could play a major role identifying subclinical inflammation and damage that had already occurred. There are small sample-sized studies with short-term follow-ups that suggest the prediction of PsA using imaging tools which needs to be tested in larger series, longer follow-ups, and by linking to other biomarkers to accurately classify pre-clinical phases of PsA and to identify which lesions are specifically associated with higher risk of arthritis development [3, 15••]. In conclusion, in the near future, the correct identification of psoriasis patients with higher risk for PsA transition could modify the management of psoriatic disease, leading to treatment strategy bridging therapy and prevention.

## References

[1] van Steenbergen HW, Aletaha D, Beaart-van de Voorde LJJ, Brouwer E, Codreanu C, Combe B (2017). EULAR definition of arthralgia suspicious for progression to rheumatoid arthritis. Ann Rheum Dis.

[2] Ogdie A (2017). The preclinical phase of PsA: a challenge for the epidemiologist. Ann Rheum Dis.

[3] Tinazzi I, McGonagle D, Biasi D, Confente S, Caimmi C, Girolomoni G, Gisondi P (2011). Preliminary evidence that subclinical enthesopathy may predict psoriatic arthritis in patients with psoriasis. J Rheumatol.

[4] Aydin SZ, Ash ZR, Tinazzi I, Castillo-Gallego C, Kwok C, Wilson C (2013). The link between enthesitis and arthritis in psoriatic arthritis: a switch to a vascular phenotype at insertions may play a role in arthritis development. Ann Rheum Dis.

[5] Wilson FC, Icen M, Crowson CS, McEvoy MT, Gabriel SE, Kremers HM (2009). Incidence and clinical predictors of psoriatic arthritis in patients with psoriasis: a population-based study. Arthritis Rheum.

[6] Ash ZR, Tinazzi I, Gallego CC, Kwok C, Wilson C, Goodfield M, Gisondi P, Tan AL, Marzo-Ortega H, Emery P, Wakefield RJ, McGonagle D, Aydin SZ (2012). Psoriasis patients with nail disease have a greater magnitude of underlying systemic subclinical enthesopathy than those with normal nails. Ann Rheum Dis.

[7] Jacques P, Lambrecht S, Verheugen E, Pauwels E, Kollias G, Armaka M (2014). Proof of concept: enthesitis and new bone formation in spondyloarthritis are driven by mechanical strain and stromal cells. Ann Rheum Dis.

[8] Schett G, Lories RJ, D’Agostino M-A, Elewaut D, Kirkham B, Soriano ER (2017). Enthesitis: from pathophysiology to treatment. Nat Rev Rheumatol.

[9] Gladman DD, Antoni C, Mease P, Clegg DO, Nash P (2005). Psoriatic arthritis: epidemiology, clinical features, course, and outcome. Ann Rheum Dis.

[10] Christophers E, Barker JNWN, Griffiths CEM, Daudén E, Milligan G, Molta C, Sato R, Boggs R (2010). The risk of psoriatic arthritis remains constant following initial diagnosis of psoriasis among patients seen in European dermatology clinics. J Eur Acad Dermatol Venereol JEADV.

[11] Eder L, Haddad A, Rosen CF, Lee K-A, Chandran V, Cook R (2016). The incidence and risk factors for psoriatic arthritis in patients with psoriasis: a prospective cohort study. Arthritis Rheumatol Hoboken NJ.

[12] Love TJ, Zhu Y, Zhang Y, Wall-Burns L, Ogdie A, Gelfand JM, Choi HK (2012). Obesity and the risk of psoriatic arthritis: a population-based study. Ann Rheum Dis.

[13] Myers A, Kay LJ, Lynch SA, Walker DJ (2005). Recurrence risk for psoriasis and psoriatic arthritis within sibships. Rheumatol Oxf Engl..

[14] Eder L, Polachek A, Rosen CF, Chandran V, Cook R, Gladman DD (2017). The Development of Psoriatic Arthritis in Patients With Psoriasis Is Preceded by a Period of Nonspecific Musculoskeletal Symptoms: A Prospective Cohort Study. Arthritis Rheumatol Hoboken NJ.

[15] Zabotti A, McGonagle DG, Giovannini I, Errichetti E, Zuliani F, Zanetti A (2019). Transition phase towards psoriatic arthritis: clinical and ultrasonographic characterisation of psoriatic arthralgia. RMD Open.

[16] Scher JU, Ogdie A, Merola JF, Ritchlin C (2019). Preventing psoriatic arthritis: focusing on patients with psoriasis at increased risk of transition. Nat Rev Rheumatol.

[17] Zabotti A, Bandinelli F, Batticciotto A, Scirè CA, Iagnocco A, Sakellariou G (2017). Musculoskeletal ultrasonography for psoriatic arthritis and psoriasis patients: a systematic literature review. Rheumatol Oxf Engl.

[18] Simon D, Kleyer A, Faustini F, Englbrecht M, Haschka J, Berlin A (2018). Simultaneous quantification of bone erosions and enthesiophytes in the joints of patients with psoriasis or psoriatic arthritis - effects of age and disease duration. Arthritis Res Ther.

[19] Naredo E, Moller I, de Miguel E, Batlle-Gualda E, Acebes C, Brito E (2011). High prevalence of ultrasonographic synovitis and enthesopathy in patients with psoriasis without psoriatic arthritis: a prospective case-control study. Rheumatol Oxf.

[20] Zuliani F, Zabotti A, Errichetti E, Tinazzi I, Zanetti A, Carrara G (2018). Ultrasonographic detection of subclinical enthesitis and synovitis: a possible stratification of psoriatic patients without clinical musculoskeletal involvement. Clin Exp Rheumatol.

[21] Dey AK, Joshi AA, Chaturvedi A, Lerman JB, Aberra TM, Rodante JA (2017). Association Between Skin and Aortic Vascular Inflammation in Patients With Psoriasis: A Case-Cohort Study Using Positron Emission Tomography/Computed Tomography. JAMA Cardiol.

[22] Maroules CD, Rosero E, Ayers C, Peshock RM, Khera A (2013). Abdominal aortic atherosclerosis at MR imaging is associated with cardiovascular events: the Dallas heart study. Radiology..

[23] Naredo E, Möller I, Corrales A, Bong DA, Cobo-Ibáñez T, Corominas H (2013). Automated radiofrequency-based US measurement of common carotid intima-media thickness in RA patients treated with synthetic vs synthetic and biologic DMARDs. Rheumatol Oxf Engl..

[24] Faustini F, Simon D, Oliveira I, Kleyer A, Haschka J, Englbrecht M, Cavalcante AR, Kraus S, Tabosa TP, Figueiredo C, Hueber AJ, Kocijan R, Cavallaro A, Schett G, Sticherling M, Rech J (2016). Subclinical joint inflammation in patients with psoriasis without concomitant psoriatic arthritis: a cross-sectional and longitudinal analysis. Ann Rheum Dis.

[25] •• Kampylafka E, Simon D, d’Oliveira I, Linz C, Lerchen V, Englbrecht M, et al. Disease interception with interleukin-17 inhibition in high-risk psoriasis patients with subclinical joint inflammation-data from the prospective IVEPSA study. Arthritis Res Ther. 2019;21(1):178 **This study suggests that very early disease interception in PsA is possible leading to a comprehensive decline in skin and subclinical inflammation**. 10.1186/s13075-019-1957-0 PMC6659205 31349876

[26] McGonagle D, Lories RJU, Tan AL, Benjamin M (2007). The concept of a “synovio-entheseal complex” and its implications for understanding joint inflammation and damage in psoriatic arthritis and beyond. Arthritis Rheum.

[27] McGonagle DG, Helliwell P, Veale D (2012). Enthesitis in psoriatic disease. Dermatology.

[28] Zabotti A, Salvin S, Quartuccio L, De Vita S (2016). Differentiation between early rheumatoid and early psoriatic arthritis by the ultrasonographic study of the synovio-entheseal complex of the small joints of the hands. Clin Exp Rheumatol.

[29] Zabotti Alen, Errichetti Enzo, Zuliani Francesca, Quartuccio Luca, Sacco Stefania, Stinco Giuseppe, De Vita Salvatore (2018). Early Psoriatic Arthritis Versus Early Seronegative Rheumatoid Arthritis: Role of Dermoscopy Combined with Ultrasonography for Differential Diagnosis. The Journal of Rheumatology.

[30] Gutierrez M, Filippucci E, Salaffi F, Di Geso L, Grassi W (2011). Differential diagnosis between rheumatoid arthritis and psoriatic arthritis: the value of ultrasound findings at metacarpophalangeal joints level. Ann Rheum Dis.

[31] Zabotti Alen, Idolazzi Luca, Batticciotto Alberto, De Lucia Orazio, Scirè Carlo Alberto, Tinazzi Ilaria, Iagnocco Annamaria (2017). Enthesitis of the hands in psoriatic arthritis: an ultrasonographic perspective. Medical Ultrasonography.

[32] Tinazzi I, McGonagle D, Zabotti A, Chessa D, Marchetta A, Macchioni P (2018). Comprehensive evaluation of finger flexor tendon entheseal soft tissue and bone changes by ultrasound can differentiate psoriatic arthritis and rheumatoid arthritis. Clin Exp Rheumatol.

[33] Cambré I, Gaublomme D, Burssens A, Jacques P, Schryvers N, De Muynck A (2018). Mechanical strain determines the site-specific localization of inflammation and tissue damage in arthritis. Nat Commun.

[34] Zhou W, Chandran V, Cook R, Gladman DD, Eder L (2019). The association between occupational-related mechanical stress and radiographic damage in psoriatic arthritis. Semin Arthritis Rheum.

[35] Tinazzi I, McGonagle D, Aydin SZ, Chessa D, Marchetta A, Macchioni P (2018). “Deep Koebner” phenomenon of the flexor tendon-associated accessory pulleys as a novel factor in tenosynovitis and dactylitis in psoriatic arthritis. Ann Rheum Dis.

[36] Aydin Sibel Zehra, Castillo-Gallego Concepción, Ash Zoe R., Marzo-Ortega Helena, Emery Paul, Wakefield Richard J., Wittmann Miriam, McGonagle Dennis (2012). Ultrasonographic Assessment of Nail in Psoriatic Disease Shows a Link between Onychopathy and Distal Interphalangeal Joint Extensor Tendon Enthesopathy. Dermatology.

[37] Tan AL, Fukuba E, Halliday NA, Tanner SF, Emery P, McGonagle D (2015). High-resolution MRI assessment of dactylitis in psoriatic arthritis shows flexor tendon pulley and sheath-related enthesitis. Ann Rheum Dis.

[38] Klingberg E, Bilberg A, Björkman S, Hedberg M, Jacobsson L, Forsblad-d’Elia H (2019). Weight loss improves disease activity in patients with psoriatic arthritis and obesity: an interventional study. Arthritis Res Ther.

[39] Aydin SZ, Can M, Alibaz-Oner F, Keser G, Kurum E, Inal V, Yazisiz V, Birlik M, Emmungil H, Atagunduz P, Direskeneli H, McGonagle D, Pay S (2016). A relationship between spinal new bone formation in ankylosing spondylitis and the sonographically determined Achilles tendon enthesophytes. Rheumatol Int.

[40] Bandinelli F, Milla M, Genise S, Giovannini L, Bagnoli S, Candelieri A (2011). Ultrasound discloses entheseal involvement in inactive and low active inflammatory bowel disease without clinical signs and symptoms of spondyloarthropathy. Rheumatol Oxf Engl..

[41] Mielants H, Veys EM, Cuvelier C, de Vos M (1988). Ileocolonoscopic findings in seronegative spondylarthropathies. Br J Rheumatol.

[42] Scarpa R, Manguso F, D’Arienzo A, D’Armiento FP, Astarita C, Mazzacca G (2000). Microscopic inflammatory changes in colon of patients with both active psoriasis and psoriatic arthritis without bowel symptoms. J Rheumatol.

[43] Cuthbert RJ, Fragkakis EM, Dunsmuir R, Li Z, Coles M, Marzo-Ortega H (2017). Brief report: group 3 innate lymphoid cells in human Enthesis. Arthritis Rheumatol Hoboken NJ..

[44] • Solmaz D, Ehlebracht A, Karsh J, Bakirci S, McGonagle D, Aydin SZ. Evidence that systemic therapies for psoriasis may reduce psoriatic arthritis occurrence. Clin Exp Rheumatol. 2020;38(2):257–61. **This study highlights the role of systemic treatment for psoriasis in the risk of PsA development.** 31287403

[45] Savage Laura, Goodfield Mark, Horton Laura, Watad Abdulla, Hensor Elizabeth, Emery Paul, Wakefield Richard, Wittmann Miriam, McGonagle Dennis (2019). Regression of Peripheral Subclinical Enthesopathy in Therapy‐Naive Patients Treated With Ustekinumab for Moderate‐to‐Severe Chronic Plaque Psoriasis: A Fifty‐Two–Week, Prospective, Open‐Label Feasibility Study. Arthritis & Rheumatology.

[46] Moll JM, Wright V (1973). Psoriatic arthritis. Semin Arthritis Rheum.

[47] Khan M, Schentag C, Gladman DD. Clinical and radiological changes during psoriatic arthritis disease progression. J Rheumatol. 2003;30(5):1022–6. 12734899

[48] Aydin SZ, Kilic L, Kucuksahin O, Ureyen SB, Kalyoncu U (2017). Performances of inflammatory back pain criteria in axial psoriatic arthritis. Rheumatol Oxf Engl.

[49] Yap KS, Ye JY, Li S, Gladman DD, Chandran V (2018). Back pain in psoriatic arthritis: defining prevalence, characteristics and performance of inflammatory back pain criteria in psoriatic arthritis. Ann Rheum Dis.

[50] Feld J, Chandran V, Haroon N, Inman R, Gladman D (2018). Axial disease in psoriatic arthritis and ankylosing spondylitis: a critical comparison. Nat Rev Rheumatol.

[51] Aydin SZ, Kucuksahin O, Kilic L, Dogru A, Bayindir O, Ozisler C (2018). Axial psoriatic arthritis: the impact of underdiagnosed disease on outcomes in real life. Clin Rheumatol.

[52] Bakirci Ureyen S, Ivory C, Kalyoncu U, Karsh J, Aydin SZ. What does evidence-based medicine tell us about treatments for different subtypes of psoriatic arthritis? A systematic literature review on randomized controlled trials. Rheumatol Adv Pract. 2018;2(1):rkx019. 10.1093/rap/rkx019 PMC6649907 31431950

[53] •• Aydin SZ, Bakirci S, Kasapoglu E, Castillo-Gallego C, Alhussain FA, Ash ZR, et al. The relationship between physical examination and ultrasonography for large entheses is best for the Achilles tendon and patellar tendon origin. J Rheumatol. 2019. 10.3899/jrheum.190169. **This study highlights the relationship between clinical and sonographic findings for large entheses.** 10.3899/jrheum.190169 31474590

[54] Solmaz Dilek, Bakirci Sibel, Jibri Zaid, Sampaio Marcos, Karsh Jacob, Aydin Sibel Zehra (2020). Psoriasis is an independent risk factor for entheseal damage in axial spondyloarthritis. Seminars in Arthritis and Rheumatism.

